# Atmospheric CO_2_ concentration effects on rice water use and biomass production

**DOI:** 10.1371/journal.pone.0169706

**Published:** 2017-02-03

**Authors:** Uttam Kumar, William Paul Quick, Marilou Barrios, Pompe C. Sta Cruz, Michael Dingkuhn

**Affiliations:** 1 International Rice Research Institute (IRRI), Crop and Environmental Sciences Division (CESD), Metro Manila, The Philippines; 2 University of the Philippines Los Baños, Crop Science Cluster, College, Laguna, The Philippines; 3 Centre de Coopération Internationale en Recherche Agronomique pour le Développement (Cirad), Montpellier, France; Universidade Federal de Viçosa, BRAZIL

## Abstract

Numerous studies have addressed effects of rising atmospheric CO_2_ concentration on rice biomass production and yield but effects on crop water use are less well understood. Irrigated rice evapotranspiration (ET) is composed of floodwater evaporation and canopy transpiration. Crop coefficient Kc (ET over potential ET, or ET_o_) is crop specific according to FAO, but may decrease as CO_2_ concentration rises. A sunlit growth chamber experiment was conducted in the Philippines, exposing 1.44-m^2^ canopies of IR72 rice to four constant CO_2_ levels (195, 390, 780 and 1560 ppmv). Crop geometry and management emulated field conditions. In two wet (WS) and two dry (DS) seasons, final aboveground dry weight (agdw) was measured. At 390 ppmv [CO_2_] (current ambient level), agdw averaged 1744 g m^-2^, similar to field although solar radiation was only 61% of ambient. Reduction to 195 ppmv [CO_2_] reduced agdw to 56±5% (SE), increase to 780 ppmv increased agdw to 128±8%, and 1560 ppmv increased agdw to 142±5%. In 2013WS, crop ET was measured by weighing the water extracted daily from the chambers by the air conditioners controlling air humidity. Chamber ET_o_ was calculated according to FAO and empirically corrected *via* observed pan evaporation in chamber *vs*. field. For 390 ppmv [CO_2_], Kc was about 1 during crop establishment but increased to about 3 at flowering. 195 ppmv CO_2_ reduced Kc, 780 ppmv increased it, but at 1560 ppmv it declined. Whole-season crop water use was 564 mm (195 ppmv), 719 mm (390 ppmv), 928 mm (780 ppmv) and 803 mm (1560 ppmv). With increasing [CO_2_], crop water use efficiency (WUE) gradually increased from 1.59 g kg^-1^ (195 ppmv) to 2.88 g kg^-1^ (1560 ppmv). Transpiration efficiency (TE) measured on flag leaves responded more strongly to [CO_2_] than WUE. Responses of some morphological traits are also reported. In conclusion, increased CO_2_ promotes biomass more than water use of irrigated rice, causing increased WUE, but it does not help saving water. Comparability with field conditions is discussed. The results will be used to train crop models.

## Introduction

The current and anticipated impact of climate change and the associated increase of atmospheric CO_2_ concentration on rice production are of great economic and social importance. This is particularly true for the tropics where rice is the dominant staple crop, and for the intensified irrigated (flooded) rice ecosystems which contribute 75% to global rice production [1].

The atmospheric concentration of CO_2_ will double by the end of this century [2], and the current level of nearly 400 ppmv already represents a 43–63% increase over pre-industrial levels [2,3]. Carbon dioxide is a growth limiting resource, particularly for C3 crops like rice. [4] reported yield increase between 3 and 18%, depending on rice cultivar, in Free-Air Carbon Dioxide Enrichment (FACE) experiments in Japan, where CO_2_ concentration was increased by 200 ppmv over current levels. [5] reported an increase in rice biomass production under similar conditions. [6] observed a 12.8% grain yield increase caused by the same CO_2_ treatment in a FACE experiment in China. So far, no FACE experiments for rice have been conducted in the tropics, but there is little doubt that rising [CO_2_] will increase yield potential if water is not limiting and heat stress does not critically reduce spikelet fertility. In fact, water is crucial for rice to avoid heat damage through transpiration cooling [7], and increased [CO_2_] tends to cause warmer canopies through partial stomatal closure [8]. Rice water requirements in a changing climate are thus a major concern, both because of the need to ensure effective transpirational cooling of the canopy and because of the globally increasing scarcity of irrigation water resources.

Irrigated rice systems mainly consume water through evapotranspiration (ET) whereas percolation losses are usually small in puddled fields [9]. Evapotranspiration may decrease under higher [CO_2_] levels because it causes partial stomatal closure and thereby increases leaf transpiration efficiency (TE), which translates into improved field level water use efficiency (WUE) [10]. Although leaf TE can increase dramatically under higher ambient [CO_2_] [5,11,12,13,14], WUE is a more complex parameter that depends on leaf area dynamics and ground cover, respiration losses and crop-generated microclimate [8,10] that are not a direct function of [CO_2_]. [5] reported an increase of WUE by 19% under +200 ppmv [CO_2_], whereas water use decreased by only 9%. This effect can be expected to be variable because stomatal sensitivity to [CO_2_] in the field is highly environment dependent [10] and [CO_2_] may thus impact on biomass or water use in a variable way. Uncertainty is particularly large for tropical climates because of scarce data. Crop-level water balance data for irrigated rice under tropical, CO_2_-enriched conditions are non-existent to our knowledge—probably because water balance studies are considered most relevant for drought-prone, non-flooded systems; and also because FACE experiments for rice so far do not exist in the tropics.

Evapotranspiration is extremely variable because it is driven by the evaporative demand of the atmosphere. A commonly used estimate of this demand is potential ET, or ET_o_, as formulated by [15] to describe the ET of a short moist grass canopy at any given weather situation, and further refined for FAO as a global standard by [16]. The crop coefficient Kc, defined as a crop’s ET divided by ET_o_, is a useful parameter to estimate ET for different crops and environments. [16] proposed Kc estimates for many crops, including rice, for early, mid and late season Kc values (e.g., 1.2 for rice in midseason in the absence of water deficit). From an analytical perspective, the concept of Kc permits to normalize observed ET values against fluctuating weather situations and thus, to distinguish between meteorological and crop-related causes of variation in ET. Potential effects of variable atmospheric [CO_2_] on ET *via* crop canopy transpiration, caused by stomatal sensitivity to [CO_2_], are bound to affect Kc unless they are compensated by changes in LAI.

The present study attempted to evaluate the effect of sub- and supra-ambient [CO_2_] on the dry mater production, water use and WUE of rice canopies in sunlit but closed chambers. The concepts of ET_o_ and Kc, which were originally designed for field crops and weather data obtained from weather stations not located within the field, were adapted for the purpose. Specifically, the study tested the hypothesis that the increased biomass production and TE of rice under super-ambient atmospheric [CO_2_] would be accompanied by reduced water requirements not only at the leaf level but also at plant population level under field-like cultivation. This information is needed to parameterize the water use algorithms of crop models for the prediction of climate change impacts on tropical irrigated rice.

## Materials and methods

### Experiments

The main study on water use and biomass production was conducted in naturally sunlit, CO_2_ controlled, temperature and humidity adjusted growth chambers during wet season of 2013 (2013 WS) at the International Rice Research Institute (IRRI) in Los Baños, Philippines. The same experiment was also conducted in the 2011 DS, 2011 WS and 2012 WS but only phenology and final crop aboveground dry weight (agdw) are reported here. The seasons DS and WS refer to calendar periods and had no effect on water resources or humidity in the chambers, but were associated with different solar radiation (Table 1). Each of four chambers corresponded to one [CO_2_] treatment (195 ppmv, 390 ppmv [current ambient], 780 ppmv and 1560 ppmv) and had a 1.44-m^2^ planted area. The semidwarf (100–105 cm maximal plant height), high-tillering, high-yielding, short to medium duration (ca. 110–115 d seed to seed), indica rice variety IR72 was grown as a transplanted (14 d after sowing) as a continuously flooded crop. The crop was exposed to the CO_2_ treatment from sowing to physiological maturity, except 1 h at pre-dawn and 1 h at post-dusk each day to flush out trace gases.

**Table 1:** Sowing date and mean daily solar radiation (Rs) at canopy tops in the chambers; for four CO_2_ concentrations and two dry seasons (DS) and two wet season (WS).

CO_2_ concentration(ppmv)	Season	Sowing date	Mean Rs, sowing to maturity(MJ m^-2^ d^-1^)
**195**	**2011 DS**	25/01/2011	11.8
**390**	**2011 DS**	25/01/2011	11.9
**780**	**2011 DS**	25/01/2011	11.9
**1560**	**2011 DS**	25/01/2011	11.8
**195**	**2011 WS**	26/08/2011	-
**390**	**2011 WS**	26/08/2011	9.3
**780**	**2011 WS**	26/08/2011	9.3
**1560**	**2011 WS**	26/08/2011	9.3
**195**	**2012 DS**	16/02/2012	13.1
**390**	**2012 DS**	16/02/2012	13.1
**780**	**2012 DS**	16/02/2012	13.0
**1560**	**2012 DS**	16/02/2012	13.1
**195**	**2013 WS**	09/09/2013	9.0
**390**	**2013 WS**	09/09/2013	9.0
**780**	**2013 WS**	09/09/2013	8.9
**1560**	**2013 WS**	09/09/2013	9.0

### Technical setup and environment control

Dimensions of walk-in growth chambers were 2.01 m (W) x 2.41 m (L) x 1.96 m (H), with a 1.2 m (W) x 1.2 x (L) 0.56 m (H) metal basin placed at its bottom to receive soil and plants (S1 Picture). The chambers were covered with Mylar (polyethylene) transparent plastic sheeting on all sides and all environment control equipment was installed inside the chamber to one side of the basin, thus limiting shading to one side only. The chambers were located in a large greenhouse hangar having glass walls on all except one side, which was on the same side at that where the equipment was installed inside the chambers. At least 3.5 m free space was provided around each chamber in all directions to permit maximal lateral illumination. Daily average solar radiation levels were 61% of that outside the greenhouse. The greenhouse structure thereby intercepted 27% of ambient solar radiation, and the chamber structure 17% of the remaining solar radiation in the greenhouse. Air within chambers was mixed with two fan systems located above canopy tops to the side of the planted plot, aspiring air from blow and blowing it against the chamber ceiling on top of the planted area. This caused a turbulent circulation from bottom to top in the non-planted sector and from top to bottom through the plant canopy. A second air circuit was used to pass air through an activated charcoal filter to absorb air contaminants.

Carbon dioxide was scrubbed/injected as controlled with an infrared gas analyzer. Temperature was set to 27°C day and 25°C night (cooling only) and relative humidity (RH) to about 75%. The system was not always able to maintain these values at midday, resulting in the mean Tmax and RHmin values presented in Table 2. Global radiation inside the chamber was recorded with a Davis^™^ weather station. Daily maximum temperature (Tmax), minimum temperature (Tmin), photosynthetically active radiation (PAR) and relative humidity (RH) were recorded with PT100 (T), LiCor Line Quantum Sensor (PAR) and EE16 (RH) sensors. Wind speed could not be measured reliably because of the turbulent air movement. Condensation water from the cooling and humidity control system was collected and quantified with a tipping bucket gauge.

**Table 2:** Summary of mean climate variables inside the CO_2_ chambers observed during the crop cycle in WS 2013 on top of the crop canopy.

CO_2_ chamber	Tmax (°C)		Tmin (°C)		RHmin (%)		Rg (MJ m^-2^ d^-1^)	
	Mean	Stdev	Mean	Stdev	Mean	Stdev	Mean	Stdev
195 ppm	28.6	1.1	24.1	0.6	62.0	7.5	8.95	3.31
390 ppm	28.7	1.1	24.1	0.4	68.4	8.1	8.99	3.32
780 ppm	29.1	1.2	22.8	1.2	68.7	8.9	8.95	3.31
1560 ppm	28.5	1.1	23.9	0.4	73.2	8.0	8.98	3.31

### Crop management and sampling

The crop culture basins were filled with puddled topsoil from IRRI paddy rice fields to a depth of 0.5 m and were irrigated to maintain 3–5 cm standing water throughout the crop cycle, causing anaerobic conditions. IR72 pre-germinated seed was sown onto flat seedling nursery trays and grown for 14 d inside the chambers, then transplanted as single seedlings at 20 cm x 20 cm spacing. Sowing for 2013 WS was on 09 September.

Weeds were managed by hand picking and insects were managed by spraying recommended pesticides as needed. The plants were fertilized with 135.7–121.8–345.1–10.15 kg ha^-1^ N, P, K and Zn respectively. P, K, and Zn fertilizers were applied as basal dose before transplanting and N fertilizer (as urea) was split (16% applied at 7–8 days after transplanting (DAT), 52% at 35–56 DAT, 25% at 63–84 DAT and 7% at 91–99 DAT.

#### Measurements

*Canopy growth*: Leaf area measurements were done by two methods, at 81 DAS (about flowering) a non-destructive measurement around midday using Accupar LP-80 (Decagon Inc., Pullman, WA, USA) light interceptor system with the extinction coefficient set to 0.6; and at physiological maturity (PM) destructive measurement of physical leaf area using LiCor-3100 (LiCor, Nebraska, USA).

*Leaf gas exchange*: a LI-6400XT portable photosynthesis system (LiCor, Nebraska, USA) was used between 29 October and 02 November 2013 (51 DAS to 55 DAS) while setting the instrument to the chamber’s CO_2_ concentration and uniform RH and T settings at saturating PAR (observed air temperature in cuvette: 27.3°C at 195 ppmv, 27.7°C at 390 ppmv, 27.7°C at 780 ppmv, 28.0°C at 1560 ppmv); block temperature at 30°C; RH at 70%; photosynthetically active radiation at 1500 μmol photons m^-2^ s^-1^). Only transpiration efficiency (TE) is presented in this paper.

*Evapotranspiration (ET)*: The condensing water from the air conditioners was trapped and measured by tipping bucket rain gauges (Model; TR-525M, 25 mm collector, Metric. Texas Electronics, Inc. 5529 Redfield Street, Dallas, TX 75235, USA) on a subsample of days (Table 3). Based on the daily amount of water collected and the cropped surface area, ET (mm d^-1^) was calculated.

**Table 3:** Daily measured evapotranspiration (ET), calculated potential evapotranspiration (ET_o_), and derived crop coefficient (K_C_ = ET ETo-1) for 27 days in the growth chambers having different CO_2_ concentration.

Days after sowing(DAS)	Evapotranspiration (ET), observed [mm d^-1^]				Potential evapotranspiration (ET_o_), [mm d^-1^]				Crop coefficient K_C_			
	195 ppmv	390 ppmv	780 ppmv	1560 ppmv	195 ppmv	390 ppmv	780 ppmv	1560 ppmv	195 ppmv	390 ppmv	780 ppmv	1560 ppmv
22	2.54	2.78	4.61	3.23	2.23	2.01	2.07	2.06	1.14	1.38	2.23	1.57
23	2.94	1.8	5.88	3.61	2.99	2.82	2.90	2.85	0.98	0.64	2.03	1.27
24	2.9	3.43	5.23	3.81	2.71	2.51	2.51	2.53	1.07	1.36	2.08	1.51
28	2.05	2.13	3.51	2.56	3.32	3.19	3.25	3.12	0.62	0.67	1.08	0.82
29	2.08	2.11	4.69	2.7	2.54	2.46	2.34	2.40	0.82	0.86	2.01	1.13
41	3.89	4.36	9.28	5.35	3.45	3.19	3.21	3.30	1.13	1.37	2.89	1.62
68	6.38	7.75	7.67	7.47	2.85	2.73	2.76	2.63	2.24	2.84	2.77	2.84
69	6.32	6.82	7.48	7.63	2.89	2.77	2.86	2.67	2.19	2.46	2.61	2.86
70	6.29	6.04	6.67	6.61	2.80	2.53	2.63	2.50	2.24	2.39	2.54	2.64
71	3.61	3.21	4.11	4.45	1.51	1.36	1.33	1.35	2.38	2.36	3.09	3.30
72	4.4	4.97	6.52	5.43	2.26	2.07	2.01	2.01	1.94	2.40	3.25	2.70
73	6.09	6.42	8.04	7.3	2.49	2.33	2.29	2.33	2.44	2.76	3.52	3.13
78	4.66	7.15	6.58	6.54	2.04	1.80	1.85	1.63	2.28	3.97	3.56	4.01
79	3.77	6.3	6.03	6.08	1.92	1.78	1.80	1.75	1.97	3.54	3.35	3.47
80	5.5	7.5	7.85	7.63	2.70	2.55	2.62	2.54	2.04	2.95	2.99	3.00
81	3.63	5.77	6.74	6.3	1.82	1.71	1.70	1.68	2.00	3.37	3.97	3.75
84	5.03	6.52	6.91	6.59	2.44	2.26	2.27	2.13	2.06	2.88	3.04	3.09
85	3.43	4.79	4.97	5.38	1.65	1.35	1.23	1.47	2.08	3.56	4.05	3.66
86	2.49	4.79	6.07	5.32	1.76	1.67	1.59	1.48	1.41	2.86	3.82	3.59
92	5.27	2.25	6.53	6.37	2.08	1.83	1.86	1.85	2.53	1.23	3.52	3.44
95	2.82	3.28	4.51	4.58	1.96	1.72	1.83	1.75	1.44	1.90	2.47	2.62
101	4.65	4.52	7.62	5.75	1.61	1.27	1.23	1.29	2.88	3.55	6.21	4.46
106	5.45	6.22	7.41	6.26	2.26	2.14	2.00	2.04	2.41	2.91	3.70	3.07
109	5.59	5.72	6.73	5.68	1.57	1.48	1.38	1.33	3.56	3.88	4.89	4.27
110	2.51	3.58	6.57	4.2	1.52	1.25	1.42	1.26	1.65	2.85	4.64	3.33
116	3.91	4.9	6.36	5.67	2.82	2.69	2.63	2.65	1.39	1.82	2.42	2.14
117	3.96	5.64	6.87	6.02	3.21	2.94	3.02	2.85	1.23	1.92	2.28	2.11
Mean	4.15	4.84	6.35	5.5	2.35	2.16	2.17	2.13	1.86	2.40	3.15	2.79

### Calculation of potential evapotranspiration (ET_o_)

Potential evapotranspiration (ET_o_) was calculated from the climate variables in the chambers Penman-Monteith equation recommended by FAO [16], as follows:

$${\text{ET}}_{\text{o}}=\frac{0.408\Delta \left({\text{R}}_{\text{n}}-\text{G}\right)+\gamma \;\frac{900}{\text{T}+273}{\text{u}}_{2}\left({\text{e}}_{s}-{\text{e}}_{a}\right)}{\Delta +\gamma \left(1+0.34{\text{u}}_{2}\right)}$$

*Where*:

ET_o_ reference evapotranspiration [mm day^-1^],

R_n_ net radiation at the crop surface [MJ m^-2^ day^-1^],

G soil heat flux density [MJ m^-2^ day^-1^],

T mean daily air temperature at 2 m height [°C],

u_2_ wind speed at 2 m height [m s^-1^],

e_s_ saturation vapor pressure [kPa],

e_a_ actual vapor pressure [kPa],

e_s_−e_a_ saturation vapor pressure deficit [kPa],

Δ slope vapor pressure curve [kPa °C^-1^],

γ psychrometric constant [kPa °C^-1^].

The term (R_n_-G) [MJ m^-2^ d^-1^] is not commonly available but was derived for short plant canopies according to [16] from the average shortwave radiation measured with a pyranometer. Since wind speed was not measurable in the chambers due to turbulent conditions, a value was estimated empirically. The linear correlation between pan evaporation and ET_o_ data in the field as provided by the local weather station was compared was used to adjust correct chamber ET_o_ data by varying wind speed input to the Penman-Monteith equation, based on pan evaporation measured in the chambers in the presence of flooded soil but no crop. The appropriate wind speed value for the chamber to obtain the field-based ET_o_ vs. pan evaporation relationship was about 1 m s^-1^.

### Calculation of crop coefficient Kc, total cumulative ET and WUE

Daily ET_o_ values throughout the crop cycle were needed because ET was measured only on 27 days (Table 3) and had to be interpolated for the other days, in order to calculate WUE from final agdw and cumulative ET. This was done by the following steps, by using the observed dynamics of crop coefficient for evapotranspiration Kc:

1. Estimation of daily ET_o_ for all days of the crop cycle
2. Calculation of Kc = ET ET_o_^-1^ [16] for the days where ET was measured
3. Calculation of mean Kc for three crop development periods (22–29 DAS, early vegetative stage; 68–86 DAS, heading and flowering stages; 95–117 DAS, late maturation stage) (S1 Fig, Panel C)
4. Establishing an approximately sigmoidal, empirical growth function for Kc (S1 Fig, Panel A)
5. Establishing an approximately bell shaped, empirical, overall response function of Kc *vs*. [CO_2_] (S1 Fig, Panel B); whereby we considered Kc = 1 before crop establishment, corresponding to an open water surface
6. Establishing a combined model predicting Kc from growth stage and [CO_2_] by multiplying function (4) with function (5) (S1 Fig, Panel C), and testing its accuracy with the measured data (S1 Fig, Panel D; R^2^ = 0.96)
7. Calculating ET for each day of the growth cycle with this model for all [CO_2_] treatments

Finally, WUE was calculated by dividing final agdw by the cumulative ET, with WUE = agdw (∑ET)^-1^. For daily weather data refer to S1 Table.

### Statistical analysis

Analysis of variance (ANOVA, Type III sum of squares analysis) was conducted with XLSTAT (V2016, Addinsoft, Inc.) in conjunction with Excel V14.0 (Microsoft, Inc.). Regression analyses and curve fitting were conducted with SigmaPlot V13 (Systat Software, Inc.).

## Results

### Atmospheric CO_2_ and season effects on biomass

**Fig 1:**
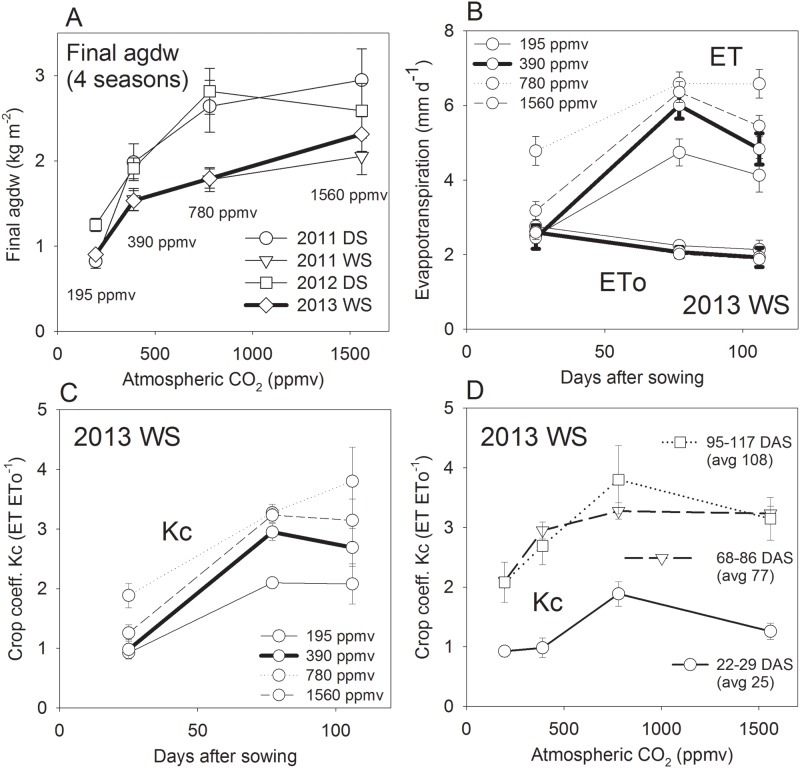
A: Response of final above ground dry weight (agdw) to atmospheric CO_2_ concentration for two dry seasons (DS) and two wet seasons (WS), differing in solar radiation levels. B: Dynamics of potential ET (ET_o_, Table 3) and crop ET in 2013 WS. C: Dynamics of crop coefficient Kc (ET/ET_o_) in 2013 WS. D: Response of Kc to atmospheric CO_2_ concentration during three periods of crop development. Error bars represent SEM for multiple measurements on different plants within a chamber.

Dry and wet seasons differed in solar radiation (Table 1) and thus gave different levels of biomass (Table 4). Final agdw at maturity responded strongly to atmospheric [CO_2_] (Fig 1A). Although the response approached saturation beyond 780 ppmv (corresponding to doubling of current levels), the current level of 390 ppmv was distinctly sub-optimal for rice biomass production. In absolute terms, final agdw observed in the chambers was similar to or slightly above the values that were independently observed in the field for the same cultivar IR72 (field data reported by [17]). Daily solar radiation (Rs) levels in the chambers averaged at 11.9, 9.3, 13.0 and 9.0 MJ m^-2^ d^-1^ for 2011 DS, 2011 WS, 2012 DS and 2013 WS, respectively, representing 61% of those in the field (details in Table 1). Consequently, the chamber crops produced similar biomass as field crops in comparable seasons [17], but with 39% less solar radiation.

**Table 4:** Observations and ANOVA for measured days from sowing to flowering, days from sowing to grain maturity and aboveground dry weight (agdw) for four CO_2_ concentration treatments and four seasons.

CO_2_ treatment [ppmv]	Season	Measured variables			
		Flowering [d]	Maturity [d]	agdw [g m^-2^]	
195	2011 DS	81	125	822	
195	2011 WS	n.a.	n.a.	n.a.	
195	2012 DS	75	106	1248	
195	2013 WS	83	120	900	
390	2011 DS	74	123	1984	
390	2011 WS	84	122	1547	
390	2012 DS	75	105	1912	
390	2013 WS	73	117	1533	
780	2011 DS	79	122	2640	
780	2011 WS	84	121	1779	
780	2012 DS	76	106	2816	
780	2013 WS	81	120	1793	
1560	2011 DS	77	125	2948	
1560	2011 WS	80	120	2053	
1560	2012 DS	74	95	2587	
1560	2013 WS	73	118	2314	
**Means:**					
Mean (195 ppmv)		79.7 (+4%)	117.0 (±0%)	990 (-43%)	
Mean (390 ppmv)		76.5 (±0%)	116.8 (±0%)	1744 (±0%)	
Mean (780 ppmv)		80.0 (+5%)	117.3 (±0%)	2257 (+29%)	
Mean (1560 ppmv)		76.0 (-1%)	114.5 (-2%)	2476 (+42%)	
Mean (total)		77.9	116.3	1925	
**ANOVA:**					
Variable	Factor	SS	F	P (by factor)	P (model)
Flowering [d]	[CO_2_]	72.0	3.81	0.058	0,025
	Season	125.3	6.63	0.015	
Maturity [d]	[CO_2_]	30.6	1.15	0.386	<0.0001
	Season	1031.6	38.81	<0.0001	
agdw [g m^-2^]	[CO_2_]	4.88E+06	28.9	<0.0001	<0.0001
	Season	1.17E+06	6.9	0.013	

Between the two wet seasons and between the two dry seasons, absolute agdw and its response pattern to [CO_2_] were similar, indicating that the results were highly reproducible. Dry season crops produced greater agdw than WS crops due to greater Rs. Mean agdw across all seasons was 1744 g m^-2^ for 390 ppmv (current ambient level), and it decreased by 4456% at 195 ppmv (0.5 x ambient), increased by 29% at 780 ppmv (2 x ambient) and increased by 42% at 1560 ppmv (4 x ambient) (Table 4). The season effect on agdw was significant (P < 0.05) and the [CO_2_] effect was highly significant (P < 0.001).

### Atmospheric CO_2_ effects evapotranspiration

Potential evapotranspiration (ET_o_) was between 2 and 3 mm d^-1^, which is close to the values reported in earlier studies [18,19] for the wet season in the Philippines (Fig 1B). Crop ET increased with crop development and attained a maximum at about flowering stage, with about 6 mm d^-1^ for the 390 ppmv treatment. This translated into a crop coefficient Kc (Kc = ET ET_o_^-1^) of about 1 during seedling stage and about 3 around flowering for the 390 ppmv treatment (Fig 1C). The latter value is much higher than the standard value estimated for irrigated rice by FAO [16] and this observation will be discussed in the succeeding section.

The response of Kc to atmospheric CO_2_ concentration is shown in Fig 1D for three stages of crop development. The Kc was highest for the 780 ppmv treatment and tended to decrease at higher concentrations. Consequently, increased [CO_2_] compared to current levels (390 ppmv) increased crop water use, whereas reduced CO_2_ reduced crop water use.

### Atmospheric CO_2_ effects on phenology and some morphological parameters

Effects of [CO_2_] and season on phenology were small affecting days to flowering and days to maturity by 5% or less (Table 4). The [CO_2_] effects were non-significant (P > 0.05) but the season effect on days from sowing to maturity was highly significant despite its small magnitude (P < 0.001). Consequently, phenology did not contribute to the strong [CO_2_] effects on agdw.

**Fig 2:**
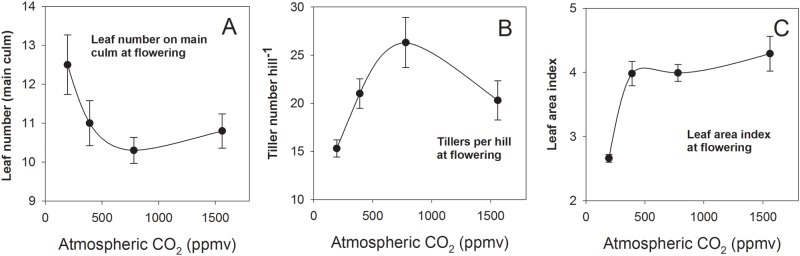
Response to atmospheric CO_2_ concentration of number of leaves appeared on main culm (A), number of tillers produced per hill (B) and leaf area index (C) at flowering stage. Error bars indicate SEM of means of biological replications within a chamber and not replications of treatment.

Number of leaves appeared on the main stem at flowering (without counting the prophyll) was 11 at 390 ppmv [CO_2_] (Fig 2A). Increased [CO_2_] had no significant effect but lower levels (195 ppmv) increased leaf number significantly (P<0.05), whereby leaves were considerably smaller (leaf size data not presented). Opposite effects were observed for tiller number at flowering (Fig 2B), which was strongly decreased at 195 ppmv [CO_2_]. It showed a bell-shaped response to CO_2_ concentration, the maximum occurring at the super-ambient concentration of 780 ppmv.

Leaf area index at flowering was in the typical of range of values found for IR72 in the field, with 6.6 at for 390 ppmv [CO_2_] and similar values at greater concentrations (Fig 2C). The sub-ambient concentration, however, strongly decreased LAI. Measurements of LAI were indirect and non-destructive, and therefore only gave trend information.

### Crop water use

In order to estimate total crop water use in the absence of ET measurements for some periods of the crop cycle (Table 3), we established an empirical relationship between Kc, DAS and atmospheric CO_2_ concentration and predicted ET with it for all days of the crop cycle (S1 Fig). The model was assembled from the mean responses of Kc-1 to DAS (S1 Fig, Panel A; 3^rd^ order power function forced through origin) and to CO_2_ concentration (S1 Fig, Panel B; 2^nd^ order). Multiplication of both models and addition of 1 (for ET_o_) gave a 3D surface of Kc response to both variables (S1 Fig, Panel C) and a good fit of calculated vs. observed Kc (S1 Fig, Panel D; R^2^ = 0.96).

**Fig 3:**
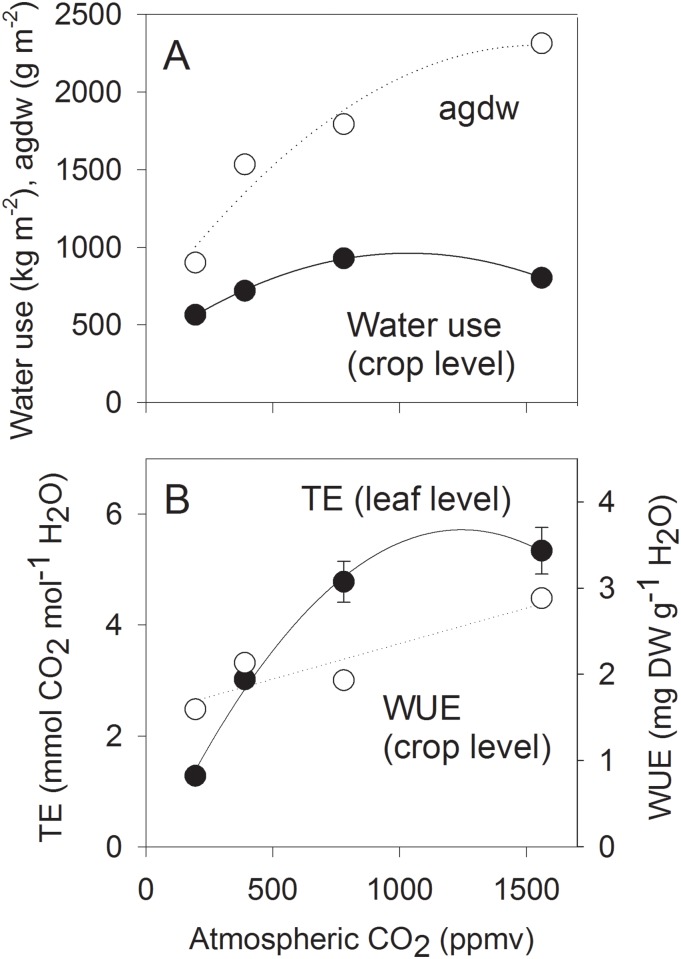
A: Response to atmospheric CO_2_ concentration of final total agdw (TDW) and cumulative crop water use. Water use was calculated from daily calculations of Kc as shown in S1 Fig. B: Response of leaf-level transpiration efficiency (TE) and crop-level water use efficiency (WUE).

Directly measured water use (in terms of ET) during the 27 days of observation scattered over the crop cycle (Table 3) was 112 (195 ppmv), 131 (390 ppmv), 171 (780 ppmv) and 149 (1560 ppmv) mm, indicating and increase by 31% when the current, ambient [CO_2_] was doubled to 780 ppmv (Table 5). Calculated total water use from sowing to maturity was 565, 719, 928 and 803 mm d^-1^ (= kg m^-2^) for 195, 390, 780 and 1560 ppmv CO_2_, respectively (Fig 3A). Doubling of current, ambient [CO_2_] increased water use by 29% (Table 5). The extrapolation of measured ET to the whole crop cycle thus conserved the proportions among treatment effects. However, the extrapolated values are more meaningful than the raw data in Table 3 because they (1) cover the complete crop cycle and (2) take into account the slight differences in the atmospheric conditions among the chambers.

**Table 5:** CO_2_ concentration effect on cumulative ET as directly measured during 27 days of observation (for daily values see Table 3) and for the whole crop cycle (extrapolated using the model in S1 Fig).

Period	Parameter	195 ppmv	390 ppmv(current)	780 ppmv	1560 ppmv
27 d (Table 3)	Cumulative ET	112 mm	131 mm	171	149
	[CO2] effect	- 14%	0	+ 31%	+ 14%
Whole cycle	Cumulative ET	565 mm	719	928 mm	803 mm
	[CO2] effect	- 21%	0	+ 29%	+ 12%

The results indicated that sub-ambient CO_2_ concentration reduced water use and super-ambient CO_2_ concentration increased it, but with a declining trend at 1560 ppmv. This declining trend was not observed for biomass, and consequently water use efficiency (WUE) increased at high CO_2_ concentration (Fig 3B).

Since only four points were available for the response of WUE to CO_2_ concentration (although based on 27 ET observations per treatment), and no error term could be calculated (because whole-cycle ET extrapolation gave a single value), the shape of the response remained uncertain. However, a linear trend with a positive slope is a plausible interpretation of the data. WUE was 1.6 g kg^-1^ at 195 ppmv [CO_2_], 2.9 g kg^-1^ at 1560 ppmv [CO_2_], and intermediate at intermediate [CO_2_]. Transpiration efficiency (TE) of the flag leaf at flowering responded much more strongly to [CO_2_] than did crop level WUE, and approached a plateau towards the highest CO_2_ concentration (Fig 3B).

## Discussion

### Biomass production

Final agdw was 1540 g m^-2^ (SE = 6; N = 2) in the WS and 1948 g m^-2^ (SE = 36; N = 2) in the DS at ambient [CO_2_] levels (390 ppmv) (Fig 1A). These values are similar to field observations for the same variety at the site, and growth duration was also near-identical [17]. Although these results may indicate that chamber conditions were representative of the field, some caution is warranted because the chamber wall and the greenhouse roof together intercepted 39% of the natural solar radiation. A lower biomass production was thus expected in the chambers, but the dimming effect was probably compensated by (1) some lateral light interception due to small plot size (1.44 m^2^) despite a 1-row planted border; (2) the higher proportion of diffuse radiation due to scattering by chamber and greenhouse wall/roof material; and (3) the highly protected conditions in the chambers.

In terms of agdw response to CO_2_, a typical asymptotic response was observed with diminishing slope as it approached saturation. The maximal agdw at saturating CO_2_ concentration (1560 ppmv) was 2183 g m^-2^ (SE = 130) in the WS and 2768 g m^-2^ (SE = 180) in the DS, or +42% in both seasons as compared to the ambient treatment. This confirmed the highly limiting nature of the carbon resource for irrigated rice, and is in line with numerous previous studies on rice [20] (review: [21]) and wheat [22,23,24]. An important validity test is the comparison with the +200 ppmv (590 ppmv) scenarios investigated in rice FACE studies in Japan and China (for comparison of sites: [25]). By interpolation, our results indicate a 22% increase in agdw for the 590-ppmv scenario, as compared to a 29% increase observed by [25] in China on average for three cultivars and a 13% increase observed by [26] in Japan for 8 cultivars. Genotypic differences were large, with indica and high-tillering cultivars responding more strongly to enhanced CO_2_ concentration. In our study the high-tillering, indica cv. IR72 was used. We conclude that the chamber-based observations on agdw response to CO_2_ are fully supported by the two FACE studies conducted on rice.

### Causes of high Kc values in chambers

The observed Kc value at 390 ppmv [CO_2_] at crop flowering (3.0) was far higher than the standard value proposed by FAO for the field (1.2; [16]). [27] observed a Kc of about 1.4 for fully developed, flooded rice canopies at the landscape scale. [28] observed a similar Kc of 1.42 at the field scale (indica materials). [10] reported a Kc of 1.24 for japonica rice in a FACE study under ambient [CO_2_], and [19]. reported even lower values. The high Kc values observed here in the chambers were probably not due to underestimations of ET_o_ because Kc values were realistic (near 1) at the beginning of the crop cycle (open water surface with no crop canopy). According to [16,29], the ET of open water surfaces is similar to that of a wet, short grass canopy and thus ET_o_, corresponding to Kc = 1.

The likely cause of the high Kc observed in mid and late season resides in the fact that Kc under field conditions is referenced by weather observed at 2m height on terrain not located in the cropped area, whereas in the chambers the temperature and humidity were measured near canopy tops. A large boundary exists between field rice canopies and weather stations. The crop develops its distinct microclimate (oasis effect) that is different from the conditions measured at a weather station, whereas in the chambers the turbulent air mixing and short physical distances provided for no such boundary. The aerodynamic coupling of the canopy to the atmosphere contributes to the magnitude of ET [30]. The absence of an oasis effect in the chambers may therefore explain the high apparent Kc but this should not cause a bias in the relative effects of the [CO_2_] treatments. FACE experiments are also affected by this problem but to a smaller extent [31,32].

The application of the ET_o_ and Kc concepts to chamber studies is obviously problematic if the objective is to derive water balance information for field extrapolation. In this study, however, the Kc concept was employed for the purpose of extrapolation of ET from the 27 observed days (Table 3) to all days of the crop cycle, in order to calculate cumulative ET and WUE. By this modeling procedure, the originally observed [CO_2_] effects on ET were conserved (Table 5), but crop ET totals were obtained, and effects of the slight differences in conditions among chambers were compensated for.

### CO_2_ effects on evapotranspiration

We observed an increase of Kc from 390 to 780 ppmv [CO_2_] (Fig 1C; S1 Fig, Panel B), followed by a decrease from 780 to 1560 ppmv. This stands in contrast with several studies reporting a decline in evapotranspiration in CO_2_-enriched crops [11,12,13,20,]. To our knowledge, [10] published the only FACE field study so far on [CO_2_] effect on rice crop ET. They found ET throughout the crop cycle to be identical between ambient and +200 ppmv [CO_2_] during early and late season, but slightly reduced at midseason when temperatures were elevated (heat sensitive japonica rices were planted). This effect reduced overall Kc from 1.24 to 1.17, as indicated by the slopes reported between ET and ET_o_. [10] conclude that although leaf gas exchange measurements consistently indicate reductions in water use under enhanced [CO_2_], effects on water use at the crop scale are much smaller and quite different.

### CO_2_ effects on TE and WUE

The stimulation of TE by increased atmospheric CO_2_ concentration has two components, a decrease in transpiration (due to partial stomatal closure) and an increase in photosynthesis. Both are linked by a physiological tradeoff, whereby the partial stomatal closure usually has the smaller contribution to TE, e.g. 20% in the case of soybean [33]. [In these studies TE, expressed as canopy CO_2_ assimilation rate over ET, is termed WUE and must not be confused with crop-level WUE which is equal to dry weight over either cumulative ET or water use.] Atmospheric CO_2_ effects on TE are much greater than those on WUE because all processes constituting TE are directly CO_2_ dependent, whereas soil/floodwater surface evaporation and plant respiration are not, but contribute to WUE. This was also the case in our study (Fig 3B).

According to the measurements on irrigated rice by [34], which have since been supported by similar reports, WUE on a grain weight basis varied seasonally between about 0.87 and 1.32 mg g^-1^, and WUE on agdw basis would be about twice as high (ca. 1.7–2.6). [Inclusion of variable percolation rates and other water losses can substantially reduce that value.] At 390 ppmv [CO_2_], we observed a similar value for WUE of about 2.0 mg g^-1^, and experimental variation of [CO_2_] made it range from 1.6 to 2.9 mg g^-1^. We did not find reports on [CO_2_] effects WUE in the agronomic sense (final biomass over either cumulative water use or cumulative ET), and even the otherwise complete mega analysis by [21] (a review of 125 studies) only reports [CO_2_] effects on TE (thereby termed “leaf-level WUE”). According to [21], TE increases by 37% for the scenario of doubled [CO_2_]. In the present study TE increased was by 58% (780 vs. 390 ppmv [CO_2_]), while the corresponding increase of WUE was only about ca. 17% (based on linear trend in Fig 3B).

### CO_2_ effects on morphology

Most CO_2_ enrichment studies reported the absence of significant effects of elevated [CO_2_] on LAI in rice [11,35,36], and also for wheat and winter barley [37]. [10] reported that LAI of rice was increased during early stages of growth but was decreased at later development stages. The meta-analysis of [21] concluded that although [CO_2_] doubling stimulates agdw by 28% and belowground dw by 42%, LAI remains constant and is associated with a modest increase in tiller number (+14%). Consequently, tillers become both more numerous and heavier, but have reduced leaf area per tiller.

The trends observed in this study support this assessment. The LAI was strongly reduced at sub-ambient [CO_2_] but supra-ambient [CO_2_] did not increase it. There were inverse effects of [CO_2_] on tiller number vs. total leaf number appeared per main culm, indicating that within the historical and anticipated ranges (represented by 195, 390 and 780 ppmv treatments), [CO_2_] stimulates tillering but reduces the number of leaves developed per tiller.

## Conclusion

This study had the objective to test the following hypothesis: Increasing atmospheric [CO_2_] reduces water requirements of irrigated rice. Water requirements of a crop in the field are commonly expressed by Kc, an approach that normalizes crop ET by the atmospheric evaporative demand ET_o_. Although the Kc measured in the confined experimental system was different from that in the field due to different boundary conditions, it was still a valid approach to normalize ET across variable atmospheric conditions and thus permitted evaluating effects of crop development stage and [CO_2_] treatments on water use. On this basis, we did confirm that increasing [CO_2_] increased leaf level TE and crop-level cumulative WUE, but absolute water use and Kc tended to increase too, and clearly did not decrease as hypothesized. This result has implications for crop water balance modeling for future climate scenarios, but needs validation at the field scale for tropical indica rice because no such data have been reported to date.

## Supporting information

S1 Picture Naturally lit, CO_2_ controlled growth chambers used in the study. (TIF)

S1 Fig Modeling of Kc. A: Dynamics of mean [Kc-1] across CO_2_ treatments described by 3^rd^-order power regression, assuming Kc = 1 in the absence of crop. B: Response of mean [Kc-1] across developmental stages described by 2^nd^-order power regression. C: Three-dimensional surface of response of calculated Kc (Kc = 1 + Eq 1 * Eq 2) vs. the predictor variables as in A and B. D: Relationship between simulated (as in C) and corresponding observed Kc.
(TIF)

S1 Table Daily weather conditions in field and CO_2_ chambers. (XLSX)

## Abbreviations

agdw: Aboveground dry weight
DS: Dry season, corresponding to December-May at the site
ET: Evapotranspiration [mm d^-1^]
ET_o_: Potential evapotranspiration according to Penman-Monteith equation [mm d^-1^]
Kc: Crop coefficient for evapotranspiration, Kc = ET / ET_o_
LAI: Leaf area index (unitless)
ppmv: Parts per million by volume for gases. Since this is a ratio, it is unitless.
TE: Transpiration efficiency at leaf level [mmol (CO_2_) mol^-1^ (H_2_O)]
WS: Wet season, corresponding to June-November at the site
WUE: Water use efficiency at crop level [mg (agdw) g^-1^ (water used)]
