# Supplementary figures and images for: Atmospheric CO2 concentration effects on rice water use and biomass production
Source: PLoS One. 2017 Feb 3;12(2):e0169706. doi: 10.1371/journal.pone.0169706 (PMC5291415; doi:10.1371/journal.pone.0169706)

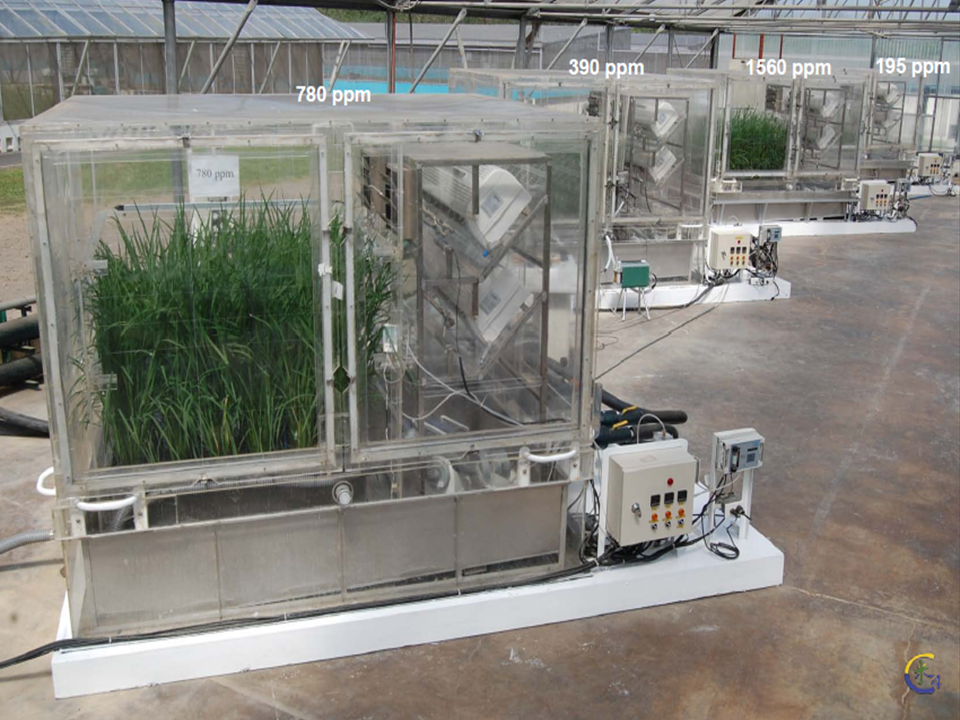

Supplement: S1 Picture — (TIF) [file pone.0169706.s001.TIF]

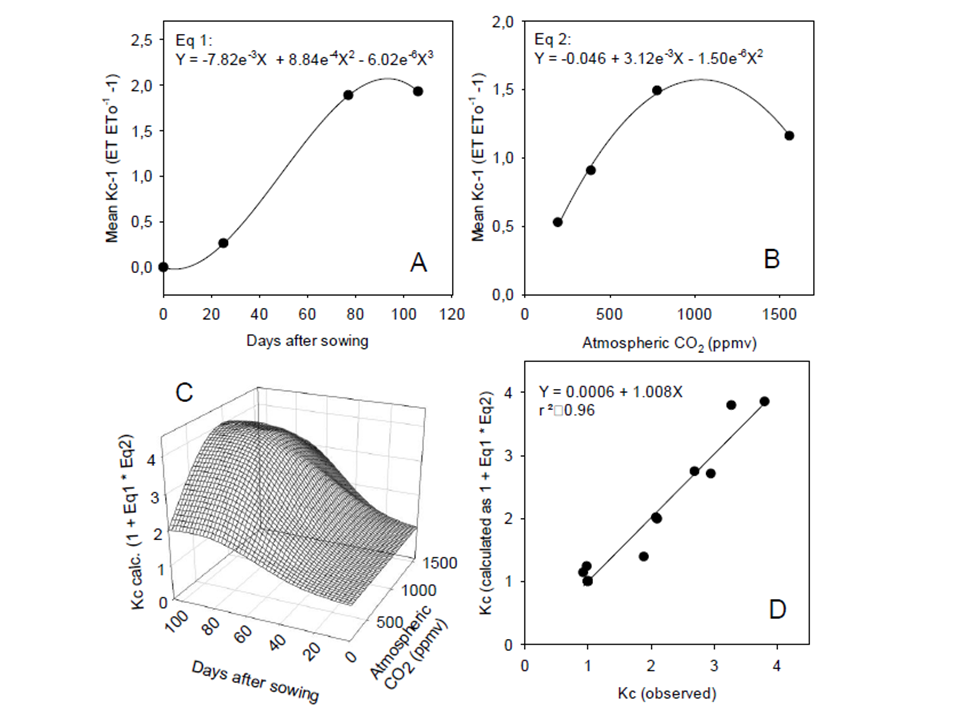

Supplement: S1 Fig — A: Dynamics of mean [Kc-1] across CO2 treatments described by 3rd-order power regression, assuming Kc = 1 in the absence of crop. B: Response of mean [Kc-1] across developmental stages described by 2nd-order power regression. C: Three-dimensional surface of response of calculated Kc (Kc = 1 + Eq 1 * Eq 2) vs. the predictor variables as in A and B. D: Relationship between simulated (as in C) and corresponding observed Kc. (TIF) [file pone.0169706.s002.TIF]
